# Greco-Arab and Islamic Herbal-Derived Anticancer Modalities: From Tradition to Molecular Mechanisms

**DOI:** 10.1155/2012/349040

**Published:** 2011-11-22

**Authors:** Hilal Zaid, Michael Silbermann, Eran Ben-Arye, Bashar Saad

**Affiliations:** ^1^Qasemi Research Center, Al-Qasemi Academy, P.O. Box 124, Baqa El-Gharbia 30100, Israel; ^2^Faculty of Arts and Sciences, Arab American University Jenin, P.O. Box 240, Jenin, Palestine; ^3^Technion—Israel Institute of Technology, Middle East Cancer Consortium, Haifa, Israel; ^4^Integrative Oncology Program, The Oncology Service, Lin Medical Center, Clalit Health Services, Western Galilee District, Haifa, Israel; ^5^Complementary and Traditional Medicine Unit, Department of Family Medicine, Faculty of Medicine, Technion-Israel Institute of Technology, Haifa, Israel and Clalit Health Services, Western Galilee District, Haifa, Israel

## Abstract

The incidence of cancer is increasing in the developed countries and even more so in developing countries parallel to the increase in life expectancy. In recent years, clinicians and researchers advocate the need to include supportive and palliative care since the establishment of the diagnosis and throughout the duration of treatment, with the goal of improving patients' quality of life. This patient-centered approach in supportive care is also shared by various traditional and complementary medicine approaches. Traditional Arab-Islamic medicine offers a variety of therapeutic modalities that include herbal, nutritional, and spiritual approaches. Physicians and scholars, such as Avicenna (980–1037), Rhazes (965–915), Al Zahrawi (936–1013), and Ibn al Nafis (1218–1288) referred to cancer etiology in various medicinal texts and suggested both preventive and therapeutic remedies to alleviate suffering. This review presents research data related to the anticancer activities of herbs used in Arab-Islamic medicine and allude to their potential role in improving the quality of life of cancer patients.

## 1. Introduction

In recent years, traditional Arab-Islamic herbal medicine has been gaining interest in the scientific community, and more specifically, regarding cancer treatment [1–3]. This school of medicine, often referred to as Greco-Arab medicine, is still influential in Arab and Islamic societies, especially throughout the Mediterranean region. Throughout Muslim history, Greco-Arab and Islamic herbal medicine were the first choice of treatment for ailments involving infertility, epilepsy, cancer, psychosomatic troubles, and depression. Arab and Muslim physicians were among the first to use scientific methods in the field of medicine, including the introduction of quantity measurements, animal testing, and clinical trials. Hospitals in the Arab-Islamic world featured drug tests, drug purity regulations, and competency tests for physicians. The earliest known tests related to public health were carried out by Rhazes (865–925), searching for the most hygienic place to build a hospital. To that purpose, he placed pieces of meat throughout Baghdad and subsequently built a hospital at the site where the meat decomposition was the least. In his *Comprehensive Book of Medicine*, Rhazes documented his own clinical cases and provided very useful documentation of various diseases. He also introduced urinalysis and stool tests. Avicenna (980–1037), who introduced quantity measurements in experimental medicine, discovered the contagious nature of diseases, introduced clinical trials, risk factor analysis, and the idea of a syndrome in the diagnosis of complex clinical entities. His book, *The Canon of Medicine*, was the first to deal with evidence-based medicine, randomized controlled trials, and efficacy tests. 

Concerning medical documentation, the first documented evidence for a peer review publication was published by Ishaq bin Ali al-Rahwi (854–931). In his work, *the Ethics of the Physician,* he stated that a physician must always document in duplicate the patient's records. When the patient was healed or died, the physician's records were examined by a local medical council, comprising of other physicians, in order to decide as to whether the treatment met the required standards of medical care. 

Cancer is a leading cause of mortality, and it strikes more than one-third of the world's population and it's the cause of more than 20% of all deaths [4]. Among the causes for cancer are tobacco smoke, viral infection, chemicals, radiation, environmental factors, and dietary factors. Contemporary physicians and biomedical researchers advocate the need for a comprehensive cancer treatment including supportive and palliative care. This patient-centered approach is based, in part, on the increased awareness of the role of traditional and complementary medicine in supportive care aimed at improving patients' quality of life. This review aims at elucidating Arab and Islamic medicines and their involvement in approaching issues associated with cancer diagnosis and treatment, while reviewing the potential role of herbs in contemporary cancer care.

## 2. Relevance of Arab and Islamic Medicine on Cancer Care in the Middle East

In early 2011, Ben-Arye and his colleagues from six Middle-Eastern countries identified 143 articles on complementary and traditional medicine that had been published in 12 Middle-Eastern countries in relation to cancer care [5]. In studies performed in Turkey and Israel, about half of the patients diagnosed with cancer reported use of complementary and alternative medicine (CAM) [6], even during chemotherapy treatment [7]. These findings are comparable with other reports in the West [8]. Herbal medicine is the leading modality used by patients with cancer in the Middle East (e.g., 35% of cancer patients using CAM in Jordan) [9] along with spiritual practices that are also prevalent (e.g., 75% of CAM users in Iranian study) [10]. CAM use is also popular among patients with pediatric [11], gynecological [12], and hematological [13] malignancies and among patients with an advanced disease [14]. Ben-Arye and colleagues reported on 59 articles published in the Middle East in relation to cancer-related herbal research including ethnobotanical surveys and reviews (7 articles), *in vitro* studies (33 articles), animal studies (8 articles), and clinical studies (11 articles) [15].

## 3. Cancer in Greco-Arab and Islamic Medicine

Roman physicians, for example, Galen (129–199), were already acquainted with tumors as clinical entities while adopting Hippocrates' (470–370 BC) basic theory of cancer being an excess of black bile. In the golden Islamic-Arab era, classic texts, including those of Galen, were translated into Arabic and thereby influenced physicians in the Arab-Islamic world. During the Golden Arab-Islamic civilization (7th to 14th century), Arab and Muslim physicians studied cancer and applied various medicines and surgical means for its treatment. In the medicinal texts of Rhazes, Avicenna, and Abulcasis, the authors distinguish clearly between the varieties of cancer types in relation to specific organs such as, eye, nasal, tongue, stomach (gastric), liver, bladder, kidney, testis, breast, spleen, and nerve tumors. Kidney cancer was first mentioned by Al Zahrawi (Abulcasis 936–1013) who was the first to differentiate between acute inflammation of the kidney and kidney cancer. Both Rhazes and Avicenna described cancer as a disease which is difficult to treat. 

Rhazes, Abulcasis, and Avicenna all realized that the prospects of curing cancer are prognostically improved if the cancer is detected at an early stage [16, 17]. Hence, the first goal of treatment would be controlling the tumor's growth. They recommended surgical removal if the tumor was small and accessible, and not close to vital organs. Avicenna (980–1037), the most influential of all Islamic philosopher scientists, suggested “*When cancer starts, it may be possible to keep it as it is, so that it will not increase and keep it non-ulcerated. It may happen sometimes that the stating cancer may be cured. But when it is advanced, verily will not*” [16, 17]. In his book, *Canon*, Avicenna described four ways to treat cancer: (a) total arrest, which was regarded as difficult; (b) preventing progress; (c) preventing ulceration; (d) treatment of the ulceration. He empathized that medications per se would not be of great value since strong medications increase “cancer evil”. In addition, “*one should avoid irritant medications. And for this, good medications are: pure minerals like washed pure tutty mixed with oils like rose oil and the oil of yellow gillyflower mixed with it”* [16]. Avicenna also described one of the very early surgical approaches for cancer treatment, as he noted “*the excision should be radical and that all diseased tissue should be removed, which included the use of amputation or the removal of veins running in the direction of the tumor … so that nothing of these will be left*” [16, 17]. He also recommended the “*use of cauterization for the area being treated if necessary.*” One mode of treatment which he discovered was the “Hindiba” (The plant *Chicorium intybus*), which Ibn al-Baitar later on identified as having anticancer properties and which could also be used for other tumors and neoplastic disorders [2, 18–20]. Avicenna had also stated that *“it (cancer) can be reached by controlling the material, improving the diet and reinforcing the involved organ by the known effective medicines, and by using mineral smears like those containing millstone dust and whet-stone dust and from smears taken from a mixture between the stone pounder for aromatics and black head stone moisturized with rose oil and coriander water…".*

## 4. Cancer Prevention and Treatment in Greco-Arab and Islamic Medicine

Based on recommendations of Rhazes and Avicenna, patients in general were treated through a scheme starting with physiotherapy and diet; if this failed, drugs were used. Rhazes treatment scheme started with diet therapy, he noted that “*if the physician is able to treat with foodstuffs, not medication, then he has succeeded. If, however, he must use medications, then it should be simple remedies and not compound ones*". Drugs were divided into simple and compound drugs. Physicians were aware of the interaction between drugs, thus, they used simple drugs first. If these failed, compound drugs consisting of two or more compounds were used. If these conservative measures failed, surgery was undertaken. 

### 4.1. Diet-Based Prevention and Therapy

Food was a substantial part of pre-Islamic medicine as well as in other traditional medicines, for example, Greek, Persian, Ayurvedic, and Chinese. Diet is a matter of faith in Islam and plays an important role in maintaining a healthy body, soul, and spirit. Muslims are commanded to follow a set of dietary laws outlined in the Holy Qur'an, where almost everything is permitted, except what Allah specifically prohibited. Later on, when the Islamic empire covered all of Arabia, half of Byzantine Asia, all of Persia, Egypt, the Maghreb (North Africa), and Spain, Arabs and Muslims not only conquered new lands, but also became exposed to foreign and multinational culinary heritages. Great developments in scientific fields, the establishment of “modern” hospitals, and growing socioeconomic conditions of Islamic empire increased the awareness of the relationship between food and health. During this period a type of Islamic food therapy was developed that was a blend of Qur'anic teaching and Greek medicine.

According to a Hadith (saying) of the Prophet, Peace Be Upon Him (PBUH) *“The stomach is the central basin of the body, and the veins are connected to it. When the stomach is healthy, it passes on its condition to veins, and in turn the veins will circulate the same and when the stomach is putrescence, the veins will absorb such putrescence and issue the same”*. Indeed, the Prophet used to recommend food for ailments even more than he prescribed herbs or medicines. The impact of diet and herbs for the well being of people was also acknowledged in the Holy Qur'an which mentioned beneficial effects of several plants and animal products on nutritional health. Among these are grapes, citrus, melon, squash, figs, dates, honey, olive oil, and black seeds. For example, figs (*Ficus carica*) are mentioned by the Prophet (PBUH) who state that “*If I had to mention a fruit that descended from paradise I would say this is it because the paradisiacal fruits do not have pits…eat from these fruits for they prevent hemorrhoids, prevent piles and help gout*." [21]. Indeed, ethnobotanical research suggests that figs are used to treat malignant and inflammatory diseases [22]. The Prophet (PBUH) also recommended the use of olive oil, by stating “*Eat olive oil and massage it over your bodies since it is a holy (Mubarak) tree”*. Dates were mentioned in twenty places in the Qur'an, as the Prophet (PBUH) was reported to have said: *“if anyone of you is fasting, let him break his fast with dates. In case he does not have them, then with water. Verily water is a purifier"* [21].

Greco-Arab and Islamic scholars, such as Avicenna, Rhazes, and Abulcasis discussed the effect of diet on cancer development and progression. While alluding to cancer prevention Avicenna quoted “*As to preventing its (cancer) progress, it can be achieved by … improving the diet and reinforcing the involved organ by the known effective medications."* This ancient attribution of the role of nutrition in cancer has been acknowledged extensively in the scientific literature involving carcinogenesis [23] and the interrelation between cancer incidence and recurrence and nutrition and lifestyle (e.g., obesity as a cancer risk factor [24, 25]).

### 4.2. Mediterranean Diet

During the last half century, epidemiological studies have consistently shown that there are clear significant positive associations between intake of fruits and vegetables and reduced rate of heart diseases mortality, common cancers, and other degenerative diseases as well as ageing. This is attributed to the fact that these foods may provide an optimal mix of dietary fiber, natural antioxidants, and other biotic compounds. Various substances in the food can control the physiological functions of the body and modulating immune responses. Immune functions are indispensable for defending the body against attack by pathogens or cancer cells and thus play a pivotal role in the maintenance of health. However, the immune functions are disturbed by malnutrition, aging, physical and mental stress, or undesirable lifestyle. Therefore, the ingestion of foods with immune-modulating activities is considered an efficient way to prevent immune functions from declining and reduce the risk of infection or cancer. 

 Traditional Mediterranean diet includes a significantly large amount and variety of plant foods, for example, fruits, vegetables, wild edible plants, breads, seeds, nuts, and olive oil. Therefore, it guarantees an adequate intake of carotenoids, vitamin C, tocopherols, *α*-linolenic acid, various important minerals, and several possibly beneficial nonnutrient substances such as polyphenols and anthocyanins and dietary fiber [26, 27].

### 4.3. Edible Wild Plants

Wild edible plants are commonly consumed in the eastern region of the Mediterranean. Wild edible herbs have always been a main part of traditional diets and were known for their health qualities among local communities and indigenous people long before their nutritious, protective, and therapeutic effects were proved by scientific research. A high percentage of individuals collect wild edible plants and consume them as part of traditional food habits. Traditional food habits have been characterized by dietary diversity and have been associated with low health risks. Wild edible plants have been identified as main components of these diets and as important contributors to their health-protective properties. Many wild species are collected from the surrounding environment and consumed as part of local diets, especially in times of shortage. Various wild greens contain high nutritional values with relatively low energy. Compared with commonly eaten vegetables, they provide the diet with greater amounts of minerals. Additionally, their antioxidant property, mainly from phytochemicals, was found to be two to three times higher than that of common vegetables [26].

### 4.4. Herbal-Based Prevention and Therapy

There is compelling evidence from epidemiological and experimental studies that highlight the importance of phytochemicals isolated from traditional medicinal plants to prevent/reduce some types of cancer and inhibit the development and spread of tumors in test animals. The term phytochemical refers to any herbal-based molecule, but in the field of diet and cancer this term is usually applied to nutritive and nonnutritive compounds that occur naturally in fruits and vegetables. More than 25% of drugs used during the last 20 years are directly derived from plants, while the other 25% are chemically altered natural products. Still, only 5–15% of the approximately 260,000 higher plants have ever been investigated for bioactive compounds. The advantage of using such compounds for cancer treatment is their relatively low/non-toxic nature [28, 29]. An ideal phytochemical is one that possesses antitumor properties with minimal side effects and has a defined mechanism of action. Some phytochemicals are likely to possess anticancer effects (Table 1). According to recent surveys, many cancer patients use complementary and alternative medicines, including phytochemicals in addition to, or following the failure of standard cancer therapy. A diet rich in fruits and vegetables has long been suggested to correlate with reduced risk of certain epithelial malignancies, including cancers in the lung, colon, prostate, oral cavity, and breast [28, 30]. Also, the cancer prevention potential of Mediterranean diets based mainly on olive tree products is known. As discussed below, the major active ingredient of the leaves and oil of *Olea europaea* is oleuropein and the majority of polyphenols found in olive oil or table olives are derived from its hydrolysis. Oleuropein is a novel, naturally occurring antioxidant compound, which may possibly be used to prevent cancer and cardiotoxicity induced by doxorubicin. Searching for medicinal benefits from edible or inedible plants is not a novel idea since numerous modern medicines have plant origins. Given that the ingestion of some plant foods results in reduced risk for cancer, researchers are delving into the identification of phytochemicals with cancer preventive ability in studies* in vitro, in vivo,* and those in humans. Phytochemicals can be roughly classified into four groups based on their mechanisms of chemopreventive and therapeutic properties, as shown in Table 1. 

In the following paragraphs, we will focus on nine widely used herbs that are commonly used in the context of cancer by patients in the Middle East: Olive, black seeds, saffron, pomegranate, nettle, Garlic, onion, Palestinian arum, and grapes [2, 3, 5, 31–33]. Other commonly used medicinal plants and wild edible plants are described in Table 1 and [30].

### 4.5. Olea Europea (Olive)

*O. europaea* (the olive) is a species of the family Oleaceae. The olive tree is an evergreen tree or shrub native to the Mediterranean, Asia, and the Maghreb region. Olive leaf and olive leaf extracts are now marketed as antioxidants, antiaging, immunostimulators, and even antibiotics. Clinical evidence has proven the antidiabetes and antihypertension effects of leaf extracts. In addition, several studies support its antibacterial, antifungal, and anti-inflammatory properties [34–36].

Epidemiological studies provide convincing evidence for a protective effect of the Mediterranean diet against cardiovascular disease and cancer [37, 38]. These findings prompted scientists to search for Mediterranean flora as a rich source of bioactive phytochemicals with a potential to evolve into preventive and possibly therapeutic agents. Much epidemiological evidence suggests that people who consume an olive oil rich diet have a lower incidence of certain cancers, including breast, skin, and colon [34]. The lower incidence of certain cancers is most likely associated with the antioxidant activity of active ingredients of the olive oil. Oxidative stress has been shown to contribute to cancer development, and antioxidants are believed to reduce the risk of mutagenesis and carcinogenesis. Hydroxytyrosol was found to be capable of protecting cells from hydrogen peroxide damage and DNA from peroxynitrite-induced damage, blocking cell cycle progression at the G1 phase, and inducing apoptosis [39]. *In vivo* and *in vitro* studies on the activity of oleuropein have found that, in addition to antioxidant properties, it has antiangiogenic action and inhibits cell growth, motility, and invasiveness. Oleuropein was also found to cause cell rounding, which disrupts the cell actin cytoskeleton. Oleuropein also affects and disrupts purified actin filaments, providing direct antitumor effects due to cell disruption. In *in vivo* animal studies, rapid tumor regression was observed when mice were given one percent oleuropein in drinking water [40]. Saturated animal fats and polyunsaturated plant fats in the diet have been implicated in colon, breast, prostate, and ovarian cancers. The vast usage of olive oil in the Mediterranean diet may explain its apparent cancer-protective effect, rather than the amount of fat consumed. Furthermore, in a recent study evaluating the antioxidant and antiproliferative activity of water and methanol olive leaves extracts in cancer and endothelial cells, olive leaf crude extracts were found to inhibit proliferation of cell from a human breast adenocarcinoma, cells from human urinary bladder carcinoma (T-24), and cells from bovine brain capillary endothelial (BBCE) [39, 41, 42].

### 4.6. Nigella Sativa (Black Seeds)

*N. sativa* of the Ranunculaceae family is one of the most commonly used medicinal plants throughout the Middle East. *N. sativa* seeds have been used for centuries as a spice and food preservative, as well as a protective and curative remedy for numerous diseases. The seeds are known to have many medicinal properties and are widely used in Greco-Arab and Islamic medicine. The plant is found wild in North Africa, the Mediterranean region, Asia Minor, and in Southern Europe. *N. sativa* is one the most referenced medicinal seeds in history. In many civilizations the herbal spice *N. sativa* was referred to as Habbat-el-barakah (literally seeds of blessing in Arabic), Kalonji (Hindi), Kezah (Hebrew), Sijah Daneh (Persian) and Black Caraway in English. The famous Greek physician dioscorides [40–42, 44, 45, 57, 59, 71–114] used black seeds to treat headaches and toothaches. *N. sativa *seeds and oil extracts have been widely used for centuries to treat disorders in the respiratory system, stomach, kidney and liver function, and circulatory, immune system as well as cancer. In Islam, it was regarded as one of the greatest forms of healing medicine available [71]. Prophet Mohammad (PBUH) stated *“The black seed can heal every disease, except death"* [21]. Avicenna referred to black seed in his “Canon of Medicine” as the seed that stimulates the body's energy and helps recovery from fatigue and dispiritedness. In the Unani Tibb system which is still practiced in central Asia and India seeds are regarded as a valuable remedy for a number of diseases. The seed's oil was used to treat skin conditions such as eczema and burns and to treat cold symptoms. 

Modern research studies showed that *N. sativa* seeds ethanol extract possesses antitumor activity in mice implanted with tumor primary cells [72]. *N. sativa* seed extracts contain amino acids, proteins, carbohydrates, alkaloids, saponins, fixed, and volatile oils. 

Thymoquinone has been found to be the main compound responsible for the pharmacological properties of the volatile oil of *N. sativa*. The biological activities and therapeutic potential of thymoquinone are discussed in details by Salem [73]. In brief, thymoquinone was found to possess potent anticancer and antioxidant abilities in animal models and cell culture systems. It acts as an antioxidant and inhibited iron-dependent microsomal lipid peroxidation, cardiotoxicity induced by doxorubin in rats, and inhibited ifosfamide-induced damage in kidney. It also prevented liver injury induced with carbon tetrachloride, lowered drug-induced toxicity and causes amelioration in the drug's anticancer activity. There are studies reporting that the anticancer potential of thymoquinone is related to its pro-oxidant activities. In human colon cancer cell cultures and in isolated rat liver mitochondria, thymoquinone induced a significant release of reactive oxygen species and inhibited the activity of aconitase, an enzyme sensitive to superoxide anion generation. One of the most promising effects of thymoquinone is its high cancer specificity and low toxicity to normal cells. This has been observed in prostate cancer, colon cancer, canine osteosarcoma, and skin cancer [74, 75]. Many multi-drug-resistant variants of human pancreatic adenocarcinoma, uterine sarcoma, and leukemia were found to be sensitive to thymoquinone [76]. These findings provide further support to the great potential of developing synthetic derivatives of thymoquinone as anticancer agents. Thymoquinone induces apoptosis through modulation of multiple targets and hence is a promising phytochemical agent that could be used for killing many types of cancer cells, such as prostate cancer cells. Thymoquinone blocked angiogenesis *in vivo*, prevented tumor angiogenesis in a xenograft human prostate cancer model in mouse, and inhibited human prostate tumor growth with almost no side effects. *In vivo*, thymoquinone inhibited the growth of prostate and colon tumors implanted in nude mice with no noticeable side effects. In colon xenografts, growth inhibition by thymoquinone was not due to decreased proliferation but rather to the significant induction of apoptosis. However, in androgen-independent prostate tumor xenografts, the suppression of tumor growth was associated with a massive apoptosis [77]. These results indicate that the antitumor activity or cell growth inhibition could in part be due to the effect of thymoquinone on cell cycle [75]. 

Although *N. sativa* seeds and oil are recognized safe, only a few studies have addressed its potential toxicity. In one study, serum gamma-glutamyl transferase and alanine aminotransferase concentrations were significantly increased after water extracted black seeds were administered orally to rats for 14 days. However, no evident pathological changes were reported [78]. Black seeds oil toxicity was tested in another study in mice and rats through examination of possible biochemical, hematological, and histopathological changes. LD_50_ was 28.8 mL/kg body for single (acute) oral determination dose and 2.06 mL/kg for intraperitoneal administration [79].

Chronic toxicity was studied in rats treated with an oral dose of 2 mL/kg daily for 12 weeks. No changes were reported neither in key hepatic enzymes levels (i.e., ALT, AST, and GSH), nor in histopathological modifications (heart, liver, kidneys, and pancreas). Nevertheless, serum cholesterol, triglyceride, and glucose levels as well as the count of leukocytes and platelets decreased significantly and slowing of body weight gain was reported. Some other studies had reported black seeds and thymoquinone toxicity in rats and mice when exposed to high doses [79–81]. Taken together, a degree of caution is necessary with larger amounts of *Nigella sativa* due to the presence of thymoquinone and other active ingredients.

### 4.7. Crocus Sativus (Saffron)

Saffron has a long history as part of traditional healing. Modern medicine has also discovered saffron as having anticarcinogenic, antimutagenic, immunomodulating, and antioxodant-like properties [82]. Saffron contains several active compounds including, but not limited to, flavonoids, tannins, carotenoids, anthocyanins, alkaloids, and saponins. A number of *in vitro* and *in vivo* studies have reported an antitumor properties of saffron [44, 82–85]. Pretreatment with saffron prevented oxidative stress induced by DMBA (7,12-dimethylbenz[*α*]anthracene), known to generate DNA-reactive species and skin carcinoma in mice [83]. These effects are contributed to an active compound, crocetin [86, 87], that exhibited antitumor activity in a lung cancer animal model by scavenging free radicals and drug metabolizing enzymes [88]. It also inhibited pancreatic cancer cell proliferation and tumor progression in a xenograft mouse model and downregulated growth and proliferation stimulated apoptosis and resulted in significant growth regression in pancreatic tumors. However, it is not known whether the effect of crocetin on pancreatic cancer regression is its own receptor-dependent or receptor-independent mechanisms [89].

Crocetins antitumor activity was evaluated in several cancer cell lines [86, 87]. For instance, crocetin inhibited MCF-7 and MDA-MB-231 breast cancer cell lines proliferation [82, 84, 90] via downregulation of matrix metalloproteinases [45]. Crocetin and carotenoids, in general, showed cytotoxic effects on a range of tumors and malignant cells [82]. It had interfered with DNA transcription as well as DNA, RNA, and protein synthesis through suppression of the activity of DNA-dependent RNA polymerase II [44]. Crocetins LD_50_ is relatively high (2 g/kg) [82, 84, 90] raising the possibility that it could be relatively nontoxic with a potential to exert an antitumor effect.

### 4.8. Punica Granatum (Pomegranate)

Pomegranates have been used for a long time in traditional Greco-Arab and Islamic medicine for the treatment of a variety of ailments, including sort throat, inflammation, and rheumatism. It was also used for treating bladder disturbances, strengthening gums, and soothing mouth ulcers. Pomegranates feature prominently in all religions: Islam, Judaism, Christianity, Buddhism, and Zoroastrianism. According to the Qur'an pomegranates grow in the gardens of paradise. Among the small number of fruits and vegetables mentioned in the Qur'an, (including date, olive, grape, banana, fig, cucumber, garlic, lentil, and onion), pomegranate was mentioned three times, indicating its significance in Muslims life. This fruit was consumed as fresh or in the form of juice. Pomegranate is known as an antioxidant agent and is used to treat several diseases including cancer, inflammation, cardiovascular disease, diabetes, bacterial infections and antibiotic resistance, and ultraviolet radiation-induced skin damage [91–93]. Yet, for the most part, research is focused on its antioxidant, anti-inflammatory, and anticarcinogenic properties.

### 4.9. Pomegranate Juice

 Pomegranate Juice is a rich source of antioxidant tannins, flavonoids (quercetin), and some other antitumor compounds. Recent research has shown that pomegranate juice selectively inhibited the growth of breast, colon, and lung cancer cells in culture, decreased proliferation and induced apoptosis of DU-145 prostate cancer cells and suppressed invasive potential of PC-3 cells. These effects may be associated with the plant-based anti-inflammatory effects [94, 95]. Pomegranate juice was also effective in inhibition of inflammatory cell signaling in colon cancer [96]. In preclinical animal studies, oral consumption of pomegranate extract inhibited growth of lung, skin, colon, and prostate tumors [95, 96]. Pomegranate fruit extract was also effective in inhibition of lung tumorigenesis in mice [97]. Thus, pomegranates consumption could potentially help in reducing the growth and spread of prostate and lung cancer cells or even prevent cancer from developing. Pomegranate juice has also shown an initial promise in a phase II clinical trial against prostate cancer [98]. Pomegranate juice given to men with rising prostate specific antigen (PSA) following surgery or radiation offered positive and beneficially significant effects on PSA parameters, suggesting a potential of pomegranate-derived products for prevention of human prostate cancer [91].

### 4.10. Pomegranate Seeds

Up to 20% of the pomegranate seed weight is oil especially fatty acids (manly is triacylglycerols) [99]. Pomegranate seeds matrix includes also lignines and some of its derivates possess antioxidant activity [100]. The seeds oil had beneficial outcome in inflammation downregulation and thus cancer preventive effects. For instance, it had inhibited PC-3 prostate cancer cell line phospholipase A2 expression [94] and upregulated MAPK-APK2 in DU-145 prostate cancer cells [57]. In mouse model, 1 *μ*g/mL seed oil suppressed tumor occurrence almost completely in mammary organ culture [101] and colon carcinogenesis induced by azoxymethane [102]. External treatment with 5% seeds oil produced significant decreases in mouse skin tumor incidence and multiplicity [103]. The oil also downregulated proangiogenic vascular endothelial growth factor (VEGF) in MCF-7 breast cancer cells and induced apoptosis in human breast cancer cells [104].

### 4.11. Pomegranate Peel

Traditionally, pomegranate peels are dried, decocted in water, and employed both internally and externally to heal aphthae and diarrhea. Pomegranate peel has been shown to possess anticancer activities, including interference with tumor cell proliferation, cell cycle, invasion, and angiogenesis [101]. Pomegranate peel extract delays the proliferation of human breast and prostate cancer cell lines [105]. Pomegranate peel and juice contain several active compounds (i.e., catechins, epicatechins, proanthocyanidins, anthocyanidins, quercetin) known to be principal for cell cycle arrest, proliferation prevention, and apoptosis initiation [106].

More specifically, catechins and epicatechins possess antiangiogenic, antioxidant, and anticarcinogenic activities [107]. They also inhibited cyclooxygenase activity, nitric oxide production, and the epidermal growth factor receptor [108].

Quercetin is well known for its anticancer activity. It had inhibited lung cancer cell growth via cell cycle arrest and apoptosis induction [109]. More recently, Park and Min showed that quercetin induces downregulation of phospholipase D1 and thus inhibited proliferation and invasion in glioma (U87) cells [110]. Quercetin anticancer beneficial effects were also evaluated in animal models [111] and in clinical trials [59]. The anticarcinogenic effects of the other isolated fractions and compounds of the pomegranate were described elsewhere [92, 112].

### 4.12. Urtica Dioica (Nettle)

The origin of its Latin name, Urtica, means “I burn”, indicative of the stings caused by glandular hairs on the leaves that contain formic acid and histamine, two agents known to cause the stinging and skin irritation after contact. *U. dioica* leaf has a long history as an herbal remedy and nutritious addition to the diet. Nettle leaves are a rich source of essential amino acids, ascorbic acid, several mineral element, and vitamins, such as iron, provitamin A, and vitamin C [113]. Nettle extracts can be used to treat arthritis, hay fever, kidney problems, pain, and anemia. Nettle extracts possess hypoglycaemic properties and improve glucose tolerance [35]. *U. dioica* is believed to be antioxidant, immunesuppressive, antirheumatoid, antiulcer, anti-inflammatory, and anticarcinogenic [14]. Indeed, its leaf [114] and roots [115] extracts were effective against prostate cancer proliferation.

### 4.13. Allium Sativum L. and Allium Cepa (Garlic and Onion)

Prophet Mohammad (PBUH) said “*although onion and garlic have a bad smell, they are cures for 70 different illnesses that cannot be cured by any other means*”. Onion (*A. cepa*) and garlic (*A. sativa*) are closely related vegetables that belong to the Allium class of bulb-shaped plants, which also includes chives, leeks, and scallions. Garlic is used for flavoring in cooking and is unique due to its high sulfur content, along with arginine, oligosaccharides, flavonoids, and selenium, all of which might promote health [116]. 

The association between the consumption of *Allium *vegetables and the risk for cancer was assessed in several epidemiologic studies, showing the protective effect of garlic and onion on cancer. In China, high consumption of *Allium* vegetables was associated with lower incidence of gastric cancer [117, 118]. Additional studies in the Netherlands suggested an inverse correlation between the risk of colorectal, breast, and lung cancers and the consumption of onion and garlic [119].

Steinmetz et al. [120] studied the association between garlic consumption and the risk of colon cancer and found that women who consumed high amounts of garlic had a 50 percent lower incidence of distal colon cancer compared with women who consumed less garlic [120]. The risk of breast cancer was also found to be reduced in women consuming greater amounts of fiber garlic and onions [31], as well as that of esophageal and stomach cancers [121]. Similar findings were noted with reference to the risk of prostate cancer [122, 123], pancreatic cancer [33], and other known cancer types [124]. The amount of garlic consumed in the above studies varied from 2 to 20 g daily (The World Health Organization (WHO) guidelines for general health promotion for adults recommend a daily dose of 2 to 5 g of fresh garlic). It was noted that although garlic had been used safely in cooking, excessive consumption can cause some side effects, in addition to those of strong breath and body odors [125]. The protective effect of *Allium* vegetables against tumor progression and against angiogenesis were attributed to its organosulfur compounds especially allicin (an active compound in garlic) and diallyl disulfide [126]. Such compounds are able to block the formation of cancer-causing substances [127], halt the activation of cancer-causing substances [128, 129], enhance DNA repair [130], reduce cell proliferation, or induce apoptosis-programmed cell death (Table 1 and [126, 131]).

The protective effects of onion and garlic organosulfur compounds against carcinogenesis were also studied in animal models and *in vitro*. When administrating the above compounds to mice 2–4 days prior to a carcinogen challenge, these compounds inhibited the development of pulmonary adenoma [132]. Intravenous administration of the garlic active compound (diallyl trisulfide) significantly retarded the growth of orthotopically transplanted hepatoma in BALB/c nude mice [133]. These compounds had also halted the proliferation of cancer cell lines, including human lung, skin and colon tumor cell lines, human neuroblastoma cells, human and murine melanoma cells, and human prostatic carcinoma cells [134–137]. 

### 4.14. Arum Palaestinum (Palestinian Arum)

Arum is edible plant and is widely used in the Middle-East cooking, especially in the Palestinian kitchen. According to a survey conducted in 2008, Palestinian Arum was found to be one of the most potent anticancerous (especially colon cancer) plant in Palestine [3]. Moreover, *A. palaestinum* is also effective against internal bacterial infections, poisoning, and disturbances of the circulatory system. Care should be taken especially when using *A. palaestinum* to treat tumor since it may cause negative side effects. For instance, flavonoid isoorientin (6-C glucoside of luteolin), isolated from *A. palaestinum* possesses myolytic activity on rat and guinea pig smooth muscle [138]. However, the action mechanism of Palestinian Arum awaits further studies.

### 4.15. Vitis Vinifera (Grapes)

Grapes exert several health benefits, including, but not limited to, anti-inflammatory and anticancer effects and prevent lipid oxidation and platelet aggregation. The main active compound in grapes is the polyphenol compound resveratrol. Resveratrol is believed to decrease circulating LDL (low-density lipoprotein) and cholesterol and thus reduce the risk of cardiovascular diseases [139]. Grapes, as many other fruits and vegetables, are rich in antioxidant compounds called flavonoids. They are among the plant chemicals that have shown a potential benefit against heart disease. Flavanoids as well as the whole black grape (including seeds) were shown to inhibit key enzymes in tumor cell, thereby inducing apoptosis and or blocking their growth [140, 141]. In rats, grape seeds extracts (proanthocyanidins) reduced the progress of ulcerative colitis thereby decreasing the risk of colorectal cancer [142].

## 5. Discussion

During the Arab-Islamic Golden Age, collaborative works of physicians and scientists from different nations and ethnic groups raised the dignity and caliber of the medical profession. Disease was seen by Arabs and Muslim physicians as a problem that can be challenged. The Prophet (PBUH) was credited with many statements on health care problems and their treatments. For instance*, “The one who sent down the disease sent down the remedy." *and *“For every disease, God has given a cure.” *He was also credited with articulating several specific medical treatments, including the use of honey, olive oil, figs, and cupping. Regarding cancer, Avicenna, Rhazes, and Al-Zahrawi have influenced the field of oncology, by establishing clinical approach and therapeutic means (i.e., surgery) which inspired medical research for five centuries. Contemporary research supports the potential of herbs used in Islamic medicine for patients with cancer. More research is warranted regarding the potential benefit of traditional Islamic herbs in alleviation of chemotherapy side effects and improved patients' quality of life. This line of research may bridge past wisdom with present needs and future perspectives, thus fostering comprehensive cancer treatment attuned to the social and religious concerns of patients allover the Middle-East.

Despite the rapidly increasing understanding of the molecular and cellular processes, the morbidity of cancer is still on the rise. Cancer epidemiology has revealed that certain cancers are more common among people of different cultures and ethnicities, such as cancer of the lung, colon, prostate, and breast, which are very common in western societies, while they are not as prevalent in eastern societies. The prevalence of cancer in the developing countries is increasing, and the global burden of cancer is estimated to approximately double between 2008 and 2030 from 12.4 million new cases per year to around 26.4 million. A majority of this increase will occur in developing countries where the health services are least able to cope with the challenge. This inequality is highlighted by the markedly lower cancer survival rates in these regions (including Arab-Islamic countries) [143], and the best way to treat cancer is by preventing it and diagnosing it at earlier stages.

Past medical literature is a valuable source of information which entails potential research topics for contemporary scientific work. Several studies have already referred to the biological activities of natural products such as stimulation of the immune system, antibacterial, antiviral, antihepatotoxic, antiulcer, anti-inflammatory, antioxidant, antimutagenic, and anticancer effects [2, 125, 144–146]. A variety of grains, cereals, nuts, soy products, olives, beverages such as tea and coffee, and spices including turmeric, garlic, ginger, black pepper, cumin and caraway confer a protective effect against cancer [31, 33, 125, 145, 147]. Several studies have also documented the relationship between decreased cancer risk and high consumption of vegetables, including cabbage, cauliflower, broccoli, brussels sprout, tomatoes, and fruits such as, apples and grapes [2, 33, 146, 148]. In addition, a number of medicinal plants and herbs have also been reported to reduce the risk of cancer in multiple sites (Table 1 and [149, 150]). With regard to anticancer drugs, various currently used drugs are the derivatives of plant sources including, but not limited to, paclitaxel (taxol), vinblastine, capsaicin, vincristine, the camptothecin derivatives, topotecan, irinotecan, and etoposide (Table 1 and [146, 151–153]). Many commonly used anticancer herbs possess chemopreventive effects within their diverse pharmacological properties. Since cancer evolves over a long period of time, agents that inhibit or retard one or more of its stages could affect the overall course of the disease. Certain micronutrients (like oleuropein and diallyl sulfide compounds found in olives and garlic, resp.) possess potent cancer-preventive capacities.

## Figures and Tables

**Table 1:** Effects of food and herbal-derived compounds in cancer chemoprevention.

Active principle(s)	Sources	Apoptosis induction	Anti angiogenesis	Anti metastasis effect	Anti inflammatory/ Antioxidant properties	References
Citral	*Aloysia citrodora* Palau*, Cymbopogon *sp.* Melissa officinalis* L.	**+**				[43]
Crocetin	*Crocus sativus *L.	**+**	**+**	**+**	**+**	[44, 45]
Curcumin	*Curcuma longa* L.	**+**	**+**		**+**	[46, 47]
Diallyl sulfide	*Allium sativum *L.	**+**	**+**		**+**	[48, 49]
Fibers, Lignans, isoflavones, and phenolic acids	*Triticum aestivum* L.	**+**				[50–52]
Several flavonoids	*Ficus carica* L.			**+**	**+**	[53, 54]
Inulin-type fructans beta(2,1) fructans	*Cichorium intybus* L.		**+**	**+**	**+**	[55]
Oleuropein	*Olea europea* L.	**+**	**+**	**+**	**+**	[39, 40, 56]
Several polyphenols	*Punica granatum *L.	**+**	**+**	**+**	**+**	[57, 58]
Quercetin	*Allium cepa *L.	**+**	**+**		**+**	[59, 60]
Silymarin	*Silybum marianum *L. Gaertn.	**+**	**+**		**+**	[61–63]
Thymoquinone	*Nigella sativa *L.	**+**	**+**	**+**	**+**	[64–66]
Several active compounds	*Arum palaestinum* L.	**+**			**+**	[2, 30, 67]
Several active compounds	Honey			**+**	**+**	[68–70]
